# Cognitive Mechanisms of Monolingual and Bilingual Children in Monoliterate Educational Settings: Evidence From Sentence Repetition

**DOI:** 10.3389/fpsyg.2020.613992

**Published:** 2021-01-20

**Authors:** Maria Andreou, Ianthi Maria Tsimpli, Elvira Masoura, Eleni Agathopoulou

**Affiliations:** ^1^ Department of Philology, University of Ioannina, Ioannina, Greece; ^2^ Department of English, School of Arts and Humanities, University of Cologne, Cologne, Germany; ^3^ Department of Theoretical and Applied Linguistics, University of Cambridge, Cambridge, United Kingdom; ^4^ Department of Developmental and School Psychology, School of Psychology, Aristotle University of Thessaloniki, Thessaloniki, Greece; ^5^ Department of Theoretical and Applied Linguistics, School of English, Aristotle University of Thessaloniki, Thessaloniki, Greece

**Keywords:** bilingualism, literacy, updating, verbal and non-verbal working memory, sentence repetition

## Abstract

Sentence repetition (SR) tasks have been extensively employed to assess bilingual children’s linguistic and cognitive resources. The present study examined whether monoliterate bilingual children differ from their monolingual (and monoliterate) peers in SR accuracy and cognitive tasks, and investigated links between vocabulary, updating, verbal and visuospatial working memory and SR performance in the same children. Participants were two groups of 35 children, 8–12 years of age: one group consisted of Albanian-Greek monoliterate bilingual children and the other of Greek monolingual children attending a monolingual-Greek educational setting. The findings demonstrate that the two groups performed similarly in the grammaticality scores of the SR. However, monolinguals outperformed the monoliterate bilinguals in SR accuracy, as well as in the visuospatial working memory and updating tasks. The findings did not indicate any bilingual advantage in cognitive performance. The results also demonstrate that updating and visuospatial working memory significantly predicted monolingual children’s SR accuracy scores, whereas Greek vocabulary predicted the performance of our monoliterate bilingual children in the same task. We attribute this outcome to the fact that monoliterate bilingual children do not rely on their fluid cognitive resources to perform the task, but instead rely on language proficiency (indicated by expressive vocabulary) while performing the SR.

## Introduction

Sentence repetition (SR) is a task that has been thoroughly used among bilingual children to assess their language level where bilinguals are assumingly weak, and cognitive resources such as working memory, where bilinguals are arguably strong. Despite the extensive use of SR with L1 children and its growing use with bilingual children, it is still unclear what exactly this task measures, i.e., whether it measures working memory ability (Baddeley, 2012) language abilities (Klem et al., 2015) and what the relationship between the two is while performing this task (Polišenská et al., 2015).

## SR and Memory

In Baddeley’s (2012) multicomponent working memory model, SR is assumed to measure the capacity of the episodic buffer, a subsystem of working memory (Alloway et al., 2004; Jefferies et al., 2004). The episodic buffer is the mediator between working memory and long-term memory and is seen as a limited capacity temporary storage system. It is responsible for integrating information from several sources to create unified memory traces, sometimes referred to as a single “episode.” In this framework, the episodic buffer is seen as a source of capacity limitation in language processing (Baddeley, 2012). A big body of literature has demonstrated that children’s performance on verbal working memory tasks correlates to their performance on SR tasks (Willis and Gathercole, 2001; Alloway and Gathercole, 2005; Baddeley et al., 2009; Riches, 2012; Poll et al., 2013; Ziethe et al., 2013; Ebert, 2014). According to Alloway and Gathercole (2005), children with good scores on a battery of simple and complex memory tasks, also demonstrated good performance on SR tasks. This link between SR and working memory can be attributed to the function of the episodic buffer as a system that integrates information from different modalities.

Within the same framework (Baddeley, 2012) simple immediate memory tasks, such as the digit span and nonword repetition, assess the ability to temporarily hold phonological material in memory, while complex tasks, such as backward digit span and listening span tasks, implicate the ability of parallel retention and processing of phonological material in memory.

## SR and Language Abilities

Considerable research has substantiated the notion that SR also taps children’s lexical knowledge (Potter and Lombardi, 1990; Stokes et al., 2006; Moll et al., 2013; Ziethe et al., 2013; Klem et al., 2015; Simón-Cereijido and Méndez, 2018). According to Klem et al. (2015), SR is best seen as a complex linguistic task that reflects the integrity of language processing systems at many different levels that is speech perception, lexical (vocabulary) knowledge, grammatical skills and speech production. In their longitudinal study with 4-to-6-year-old L1-TD Norwegian-speaking children, Klem et al. (2015) used structural equation modeling to examine the relationship between SR and other language abilities, such as vocabulary and grammatical knowledge. Their results showed that the three language skills, namely, vocabulary, grammar, and SR, defined a single latent language ability factor. This suggests that SR is best conceptualized as a measure of language ability as well as a multi-faceted task that engages virtually all aspects of language processing. In a SR task, after hearing a sentence the listener creates a conceptual (rather than form-based) representation of the sentence that is to be recalled. Having generated a conceptual representation of the sentence they have heard in order to repeat it, the listener then goes through a series of processes including activating relevant lexical (word) knowledge, grammatical encoding, and the processes involved in phonological realization and speech production. In this view, sentence repetition is seen less as a measure of memory, and particularly of the episodic buffer subsystem of working memory, and more as a measure of language abilities. With respect to bilingual language acquisition, this view is of great significance, since bilinguals present great heterogeneity with regard to Age of Onset of exposure to the L2, degree of L2 exposure, and language proficiency. These factors have been reported to have an impact on the way bilingual populations perform on SR (Chiat et al., 2013; Thordardottir and Brandeker, 2013; Meir et al., 2015). It is also evident that successful performance among bilinguals in SR in the non-dominant language requires minimal vocabulary input (Simón-Cereijido, 2017). A recent study with Spanish-English bilinguals explored the relationship between lexical measures and children’s SR performance. With 61 TD preschoolers, Simón-Cereijido and Méndez (2018) observed that a sizeable percentage of the variance in the SR scores in both English and Spanish was explained by children’s scores on an expressive vocabulary test in the language tested (54% in English and 16% in Spanish). They attributed their findings to the large role that lexical knowledge plays in performance on SR, particularly among emerging bilinguals.

## SR and Language Experience

The way bilingual children perform on any given language task is to a certain extent associated with their language experience. Despite the fact that SR tasks are supposed to be less influenced by language exposure than lexical tasks (Armon-Lotem et al., 2015), language experience is a predictive factor for children’s performance in SR in any language (Thordardottir and Brandeker, 2013). Quality of language exposure, both in terms of lexical proficiency as well as in the perception of semantic and phonemic representations of the acquired vocabulary in any of the two spoken languages of bilingual children, has an important impact not only on the ability to repeat sentences on a given SR task but also on the performance in those tasks in terms of speed.

This is even more obvious in the case of bilingual children, due to the large variability of the sample. Nevertheless, SR tasks are considered as a useful tool that has been used in order to distinguish bilingual children with typical development from children with atypical development. In particular, Pratt et al. (2020) investigated the extent to which verbal short-term memory, vocabulary, and language exposure have an influence on two SR tasks conducted both in English and in Spanish, testing 136 school-age Spanish–English bilinguals. The findings demonstrate a difference in SR performance between typically developing children and children with Developmental Language Disorder (DLD) with regard to the effect of the aforementioned skills. Furthermore, the performance of typically developing bilingual children on SR strongly correlated with their expressive vocabulary and language exposure, while the performance of bilingual children with DLD was predicted by their verbal short-term memory.

Andreou et al. (2020a) examined the effects of bilingualism in verbal and low-verbal ToM tasks. In their study, 27 monolingual and 29 bilingual children with Autism Spectrum Disorder (ASD) were tested. All children were also administered executive function and sentence repetition tasks, in order to evaluate the extent to which their performance on ToM tasks would be influenced by factors such as cognitive control and/or language ability. Their findings with regard to the SR task demonstrated that the bilingual participants scored higher than their monolingual peers in syntactically complex sentences, and more specifically in adverbial and relative clauses.

The focus of the present study centers on Albanian-Greek bilingual children monoliterate in L2 Greek, and the way they compare to a control group of monolingual Greek children, monoliterate in Greek, of the same age. This specific group is of particular interest, due to the fact that it offers the opportunity to observe the cognitive and linguistic skills of the children and how they contribute to performance in the SR task. More precisely, we investigated which variables (vocabulary or updating and WM) are stronger predictors of children’s performance on SR, with respect to monolingual vs. monoliterate bilingual children examined in the same language, Greek. In addition, we examined the extent to which monoliterate bilingual children differ from their monolingual peers in SR, in terms of accuracy and grammaticality scores in SR as well as in cognitive tasks.

## Bilingual Advantage: is it Real?

While bilingual populations have been found to fall behind when compared to their monolingual peers in some language abilities such as vocabulary, with bilingual children knowing fewer words in each language than monolingual children (Bialystok and Craik, 2010) and in naming tasks (see Bialystok et al., 2009), bilingualism has an advantageous effect on executive control systems (Bialystok and Craik, 2010), such as cognitive control, attentional control, inhibition, and switching (Bialystok, 1986). The observation that bilingual populations perform better on tasks that tap executive functions, includes better scores in working memory (Bialystok, 1999; Bialystok and Martin, 2004). These findings speak to a “bilingual advantage” and have dramatically raised research in the area. Nevertheless, several studies have questioned the “bilingual advantage” and failed to replicate the phenomenon, especially for working memory (Engel de Abreu, 2011) and other cognitive functions (Costa et al., 2009). However appealing the hypothesis that operating two language systems boosts cognitive and executive performance may be, the picture arising from recent research studies far from confirms this hypothesis, both in terms of replicability of existing research findings, as well as of other results from studies conducted during the last decade. This can be attributed to small sample sizes, biases in terms of participant selection or inappropriate methodological approaches and tools. Paap et al. (2015) conducted a review of existing literature, attempting to find robust empirical evidence toward the existence or not of a bilingual advantage in executive functions. Having examined a number of studies focusing on different behavioral and neural indicators, Paap et al. (2015) reach the conclusion that it is very difficult to detect such an advantage, and in case it does exist, it is only evident under specific circumstances. Therefore, the claim that bilingualism by itself confers an advantage should not be taken for granted. Similar outcomes are provided from the study of von Bastian et al. (2016) who tested 118 young bilingual adults in multiple executive function tasks, measuring inhibition, shifting, cognitive flexibility, and conflict monitoring when considering age of onset, language use, and proficiency. Their findings did not show any distinct bilingual advantage and they attribute possible bilingual cognitive influence on task-specific methodological approaches. A study by Hernández et al. (2013) further questioned the validity of the bilingual advantage on executive functions. They examined Catalan-Spanish bilinguals in a variety of task-switching tests and compared their performance to that of Spanish speaking monolinguals. They implemented three experiments, where they tested task restart costs, bilingual effects, and replication of low switch cost in bilinguals, as conducted in past research studies. The results of their study did not validate a bilingual advantage in low switch cost in any of the conditions, further questioning the advantage of bilingualism on executive functioning. One of the questions that remains unanswered therefore, is the identification of the factors that trigger or fail to present a bilingual advantage and the profile of the bilinguals that participate in each of these scenarios the most.

## The Role of Biliteracy in Executive Functions

One common denominator for studies on bilinguals is individual variability. A key source of individual variability relates to literacy. Research on the effects of biliteracy, i.e., the development of literacy in the two languages of a bilingual speaker, is limited compared to the wealth of research on bilingualism. A small body of studies has revealed that biliterate bilinguals outperform monoliterate bilinguals and monolinguals in phonological awareness tasks and in reading fluency measures (Leikin et al., 2010). Recently, however, the role of biliteracy has been investigated in comparison to executive function skills.

In particular, Andreou et al. (2020b) compared three groups of 8–12 year-old Albanian–Greek children who differed from each other with respect to the level of literacy in their L1 and L2. Results showed that the biliterate children performed significantly better than their monoliterate peers in tasks of nonverbal intelligence (fluid intelligence), visuospatial working memory, and verbal working memory.

In addition, Dosi et al. (2016) found that in an updating task of a cohort of 8–12 years old Greek–Albanian bilinguals drawn from the same larger research project, the biliterate bilinguals performed significantly better than the monoliterate bilinguals and that the latter group did not differ significantly from the monolingual controls. Moreover, in the same study, the monoliterate bilinguals performed worse than both the biliterate bilinguals and the monolinguals in verbal working memory, tested with a 2-back digit task.

Finally, Andreou et al. (submitted) examined the impact of bilingual education and biliteracy on executive functioning by testing 65 primary school-age Greek-German bilingual children (10–12 years old). The authors focused on differences associated with exposure to bilingual education by testing three executive function tasks of different complexity levels. Findings of a mixed-effects model demonstrated that, on one hand, age and nonverbal intelligence affected performance in the less-demanding working memory tasks, whereas balanced bilingual education had the most significant impact on performance in the most challenging task of updating. Similarly, the beneficial role of balanced educational settings on executive functions skills was also detected in Andreou and Tsimpli’s (2020) study.

## The Present Study

Within the BALED project (2012-15) “Bilingual acquisition and bilingual education: The development of linguistic and cognitive abilities in different types of bilingualism,” we assessed over 500 bilingual children, who in their majority were biliterate in L1 and L2 albeit to different degrees. There was also a smaller number of children (Albanian-Greek) growing up in Greece who had no literacy support in their mother tongue (i.e., Albanian), but who attended a monolingual and monoliterate Greek educational setting. This can be attributed to the fact that Albanian language maintenance was not of interest for many of the families of those children, due to the low prestige status the Albanian language has in Greek communities, as well as due to the lower motivation to keep ties with their country of origin for those Albanian immigrant families. Therefore, not engaging in ethnic language learning practices or even embracing the idea of bilingualism for their children was a choice for these families, that derives from the often misguided impression that the acquisition of two languages burdens the development of the dominant language of the country they live in (Chatzidaki and Maligkoudi, 2013; Kostoulas and Motsiou, 2020). Andreou (2015) examined a subgroup of the total bilingual cohort of the BALED project, i.e., 257 Greek– German, Greek– English and Greek– Albanian bilingual children. In contrast to the monolingual children that this large cohort was compared with, Andreou (2015) found that bilinguals have better executive functions and that this higher performance was driven primarily by the bilingual children’s level of biliteracy, i.e., the balance of dual literacy exposure to the bilinguals’ languages.

As already mentioned and acknowledge in the literature, bilinguals are a very heterogenous group. Because this heterogeneity may skew results on different cognitive tasks, for this study, we decided to lay our focus only on the small group of monoliterate Albanian-Greek bilinguals growing up in Greece and compare them to a control group of monolingual children. The innovation of this study lies in the selection of this particular bilingual group which includes children who demonstrate a specific set of characteristics: balance in terms of oral vocabulary skills in L1 and L2 but dominance in Greek in terms of language exposure, and literacy only in Greek L2. For more clarity and transparency, it was established that our two groups (the monolingual children and the monoliterate bilingual children) did not differ with respect to their vocabulary skills and their SES background. It should also be mentioned here that the majority of studies on SR, both in monolinguals and bilinguals, has focused on data collected from preschoolers, and therefore children who have not attended a formal educational setting as yet.

Accordingly, the research questions and hypotheses of our current study are the following:

1. Do monoliterate bilingual children differ from monolingual children in SR and cognitive skills?
Hypothesis 1. Based on our previous studies and the findings that point to the advantage of biliteracy on cognitive skills, we hypothesize that monoliterate bilingual children will perform similarly to their monolingual peers in SR and cognitive tasks (cf. Andreou, 2015; Andreou and Tsimpli, 2020; Andreou et al., 2020b). The monoliterate bilingual group in our study does not participate in bilingual education or (Albanian) heritage language support but, instead, is immersed in monolingual L2 Greek education making them dominant in L2 Greek in terms of L2 language experience. We therefore expect that no bilingual advantage will be evinced in these children’s cognitive skills. Furthermore, we expect that their performance in SR will be similar to the monolingual children’s performance provided their language proficiency measured through vocabulary skills will not be different from the monolingual children’s, on the grounds that lexical abilities contribute to performance on SR (e.g., Klem et al., 2015).
2. Which predictors (vocabulary in L2 Greek or cognitive tasks) account for the performance on SR in monolingual vs. monoliterate bilingual children?
Hypothesis 2. As indicated in the literature, several variables have been accounted for as predictors for the performance on SR, such as language experience (Armon-Lotem et al., 2015), language abilities (Klem et al., 2015) and working memory (Alloway and Gathercole, 2005; Poll et al., 2013). Based on previous studies we expect both vocabulary skills and cognitive factors to account for performance on SR. Should the two groups be similar in vocabulary skills, we hypothesize that monoliterate bilingual and monolingual children will rely on the same cognitive and linguistic mechanisms for the SR task.

## Materials and Methods

### Participants

The participants of the present study were two groups of 35 children each, namely, a group of Albanian-Greek bilingual children (mean age = 9.68 years, *SD* = 1.22 years, 20 boys), whose L1 (Albanian) literacy was not supported in any formal education context (henceforth BIL), and the Greek monolingual children (mean age = 9.66 years, *SD* = 1.13 years, 22 boys; henceforth MON). Participants were recruited from public schools in the area of Thessaloniki (Greece). The study was approved by the Research Ethics Committee of the Greek Ministry and parental consent was required for participation in the study. All participants were between 8 and 12 years old, and the two groups did not differ significantly in their chronological age, nor in their socioeconomic status (SES) as assessed by the index of maternal education (in accordance with Hoff et al., 2002; Ensminger and Fothergill, 2003) and calculated on a five-point Likert-type scale, with 5 representing the highest educational level attained, the range being from compulsory primary education to tertiary education (Mattheoudakis et al., 2016; Andreou and Tsimpli, 2020; Andreou et al., 2020b). Results indicated that the two groups did not differ significantly in non-verbal intelligence either [*t*(66) = 0.132, *p* = 0.081, *η_p_* ^2^ = 0.046].

Further information about the experience monoliterate bilingual participants had in each of the two languages was obtained through a child questionnaire (Mattheoudakis et al., 2016). It should be mentioned that participating Albanian–Greek monoliterate children came from families where both parents were Albanian. The main questions were grouped in three categories: (a) home language history, (b) current language use and (c) early literacy practices. Home language history refers to exposure to each language from birth up to the age of schooling (i.e., up to the age of 6). Current language use refers to the language preferences for daily activities (i.e., memorizing phone numbers, calculating, telling the time, or watching TV), oral interaction with family members, and friends and the language that they feel they understand or speak better. Early literacy practices refer to activities such as shared-book reading in preschool age. Following Andreou (2015) for the analysis of the questionnaire data, points were attributed for input in each language. Points were accumulated based on the number of people interacting with the child at different stages of development. For example, for home language, one or the other language was given 1 point, depending on whether a certain family member interacted with the child (father, mother, siblings, and grandparents, etc.) in Greek or Albanian, respectively. If a person interacted with the child in both languages, the point was divided between the two languages (0.5 points each). Consider, for instance, the case of a child who speaks with the father in Albanian and with the mother in Greek and Albanian. One point will be given to Albanian for the interaction with the father and 0.5 to Albanian and 0.5 to Greek based on the interaction with the mother. This score was normalized (in percentage) for the total number of persons interacting with the child (in Greek or in Albanian). Table 1 represents the percentage of the scores attributed to the Greek language. The remaining percentage corresponds to the Albanian language. Table 1 also demonstrates the age, sex and SES scores of the two groups. Continuous data in Table 1 are expressed as means and standard deviations (SDs), and the categorical data of sex and SES are presented as frequencies and percentages. Univariate tests were calculated with Mann-Whitney non-parametric tests for the continuous variables, and chi-square tests for the categorical variables. The two groups did not differ in any of the background demographic variables.

**Table 1:** Demographic characteristics of monolingual and bilingual children.

	Group means (*SD*)				*p* value^1^
	MON		BIL		
Age	9.66 (*1.13*)		9.68 (*1.22*)		0.955
Sex (Males)	20 (57%)		22 (63%)		0.832
SES	1	0	1	0	0.158
	2	3 (8.6%)	2	4 (11.0%)	
	3	22 (63.0%)	3	21 (60.0%)	
	4	8 (23.0%)	4	9 (26.0%)	
	5	2 (5.4%)	5	1 (3%)	
Home language history (in Greek)	-		63.4 (*9.8*)		-
Current language use (in Greek)	-		81.4 (*12.8*)		-
Early literacy practices (in Greek)			53.2 *(7.3)*		

MON, monolinguals; BIL, bilinguals; SD, standard deviation; SES, socio-economic status.

1 Mann-Whitney-U tests for numerical variables, chi-square tests (Yates correction) for sex.

Table 1 demonstrates that children are dominant in the L2, Greek, since even in their early literacy practices, i.e., prior to formal schooling, the language used more was Greek. As already mentioned before, this can be mainly attributed to the low prestige status that Albanian language has in Greek communities, often affecting language attitudes and practices at home. However, it should be noted that for the early literacy practices analysis of only 27 bilingual children have been included because eight of them received no early literacy practices in either Greek or Albanian.

## Assessments

The two groups (monoliterate bilinguals and monolinguals) undertook a SR task in Greek (Chondrogianni et al., 2013). Additionally, both groups were administered a task of expressive vocabulary (Vogindroukas et al., 2009) as well as tasks for nonverbal intelligence, verbal, and visuospatial working memory and updating skills. The verbal working memory task was a backward digit span task in Greek. In addition, the instructions of the non-verbal cognitive task were also presented in Greek.

### Greek Expressive Vocabulary

The Greek vocabulary test (Vogindroukas et al., 2009; adaptation from that assesses expressive vocabulary and word retrieval was administered to all participants. The test is a naming task and consists of 50 items, arranged in order of increasing difficulty. The examiner presents a picture of an object which the child is expected to name: 1 point is given for correct responses, 0 for wrong responses. Testing discontinues when the child provides wrong naming (or no response) in five consecutive trials. The highest possible score for each test is 50, with each correct naming response given 1 point (Cronbach’s alpha = 0.179).

### Nonverbal Intelligence

Children’s nonverbal intelligence was evaluated by the Raven’s Colored Progressive Matrices Test (Raven, 1995). The Raven’s Matrices tests is one of the most commonly used means of testing general intelligence in children (Bayliss et al., 2003), and loads highly on a general factor in psychometric studies of intelligence (Carroll, 1994). In this test, participants are required to complete a geometrical figure by choosing the missing piece among six possible drawings. Patterns progressively increase in difficulty. The test consisted of 36 items ordered in terms of increasing difficulty. A total overall score was calculated for each child. Given that this test had not been standardized for the Greek population at the time of data collection, the raw scores were used for the statistical analyses. The maximum score was 36 points (Cronbach’s alpha = 0.312).

### Verbal Working Memory Task

For the Backwards Digit Recall test (AWMA, Alloway, 2007) the child is required to immediately recall a sequence of spoken digits in the reverse order. The test consisted of six blocks, starting with two digits in block one, increasing to sequences of seven digits in the last block. Each correct trial was scored with a possible minimum score = 0 and maximum of 36. Digit sequences were audiotaped by a native speaker of Greek with 1-s distance between the offset of a digit and the onset of the next one. Test reliability of the AWMA is reported in Alloway (2007) and test validity in Alloway et al. (2009). This is a verbal memory span task in which the number of digits to remember increases progressively over successive blocks containing six trials each (Cronbach’s alpha = 0.317).

### Visuospatial Working Memory Task

Visuospatial working memory was assessed through the Rotating Figure task (Mr X task; Alloway 2007). In this task, the child was shown two Mr. X figures and had to identify whether they were holding the ball in the same or different hands. One Mr. X was rotated in each trial. The child then had to recall the location of the ball in Mr. X’s hand by pointing to one of eight compass points. This task aimed at tapping visuospatial working memory. The participant must simultaneously process and temporarily store visuospatial information. The participant is shown a picture of two figures called Mr. X and asked to identify whether Mr. X with the blue hat is holding the ball in the same hand as Mr. X with the yellow hat. Mr. X with the blue hat may also be rotated. At the end of each trial the child is asked to recall the location of each ball in Mr. X’s hand in sequence, by pointing to a picture with eight compass points. Each correct answer is given 1 point and each wrong answer a 0 point. For each level (span) there are six trials, which equals to six points for the corresponding number of correct answers. The first four consecutive successful trials in each level award six points and the right to move on to the second level. If the fourth correct answer is trial five, the child gets five points in total and moves to the second level, if it is trial 6 s/he gets a total of four points and moves on to the next level. The same procedure is repeated in all seven levels. The discontinuation rule applies when the child gives three wrong answers in any of the seven levels and the procedure is terminated, but in this study we use the total score on the task and not the level reached by each participant. The highest score for correct trial responses is 42, and for span is seven. The span calculation is based on the four correct responses in the last level reached by each child (Cronbach’s alpha = 0.287).

### Updating: 2-Back Task

A verbal 2-back task was used to tap children’s updating abilities. For this task children were presented with a series of letters one at a time and were asked to judge whether each stimulus matched the one presented two items previously. The interstimulus interval was a blank screen lasting 2,500 ms. Children could respond as soon as the stimulus was presented. The 2-back task requires participants to monitor the content of a temporarily presented sequence of items at a constant rate of every 4 s. The items in this test were four numbers (e.g., 2, 5, 7, and 8). The task requires participants to determine if the currently presented stimulus items matched an item that was recently presented ‘2’-back (see Picture 3). If the current digit was identical to the one presented two steps back, the participants should press “Ξ” on the keyboard. There was a practice block, followed by a test block of 60 stimuli. In this task information needs to be updated continuously to keep track of what the current stimulus must be compared against. This task consists of 60 items, 20 correct hits and 40 false alarms. There is no discontinuation rule in this task. In order to create a composite score in each participant for the N-back task, first we transformed the number of the correct hits and the number of the false alarms into percentage scores and then we subtracted the percentage of the false alarm hits from the percentage of the correct hits. An example of this measure is given: If we suppose that the correct hits are 10/20 (i.e., 50%) and the false alarms are 10/40 (i.e., 25%), then we subtract 25% from 50%, and the final score for this participant will be 25%. The score in this task could range between −100% (for the lowest score) and + 100% (for the highest score). In order to create this composite score, we took into consideration the false hits as well, since it might be possible for a participant to have 100% of correct hits and 100% of false hits, creating a total score of 0. This indicates that this specific participant pressed the “Ξ” regardless of the instructions given. Thus, we conclude that the combination of these two scores is the most appropriate way of scoring this task (Cronbach’s alpha = 0.455).

### Sentence Repetition Task

The Greek SR employed in this study was designed within the COST Action IS0804 (Chondrogianni et al., 2013). The task consisted of 32 sentences, containing a variety of structures such as negation, clitics, coordination, complement and relative clauses, wh-questions and adverbials (Kaltsa et al., 2019; Andreou et al., 2020a,b).

Procedure: During the task the child listened to each sentence only once and repeated it as accurately as possible. There was a practice session to familiarize the participants with the procedure. The participants listened to the sentences *via* headphones and their responses were recorded.

Coding: The task was assessed with respect to two factors, (a) grammaticality and (b) accuracy.

a. The grammaticality scores aimed to capture whether the utterance the participant produced was a grammatical sentence of Greek or not. One point was awarded if the sentence was grammatical, and 0 if the utterance was ungrammatical (Cronbach’s alpha = 0.307).
b. Accuracy scores aimed to capture level of accuracy in sentence repetition. If the participant’s production exactly matched the stimulus sentence, the participant received three points, whereas, if the participant made one lexical or grammatical substitution, omission or addition, they received two points. If the participant made two of the aforementioned errors, they received one point and, if the participant made three or more errors, they received no point for that sentence item (Cronbach’s alpha = 0.328).

### Albanian Expressive Vocabulary

c. The only task that was conducted in Albanian was an expressive vocabulary task (Kapia and Kananaj, 2013). This test has the same structure, procedure and scoring system as the Greek one (Min score = 0, Max score = 50). However, it should be mentioned that this tool has not been standardized for the Albanian language and in addition, it has not undergone control for whether or not it accounts for cultural biases etc. (Cronbach’s alpha = 0.304).

## Results

A series of independent samples t-tests was conducted to examine the differences between the two groups (monolingual children and monoliterate bilingual children) in all measures. The analyses revealed that the two groups did not differ in their Greek expressive vocabulary knowledge [*t*(66) = 3.033, *p* = 0.101, *η_p_* ^2^ = 0.040] nor in their nonverbal intelligence [*t*(66) = 0.132, *p* = 0.081, *η_p_* ^2^ = 0.046] (see Table 2). A separate index for bilingual children was estimated for their vocabulary in Albanian and their mean scores are reported in Table 2. With respect to the verbal working memory task, no differences were detected between the two groups [*t*(66) = 0.184, *p* = 0.854, *η_p_* ^2^ = 0.001]. In contrast, differences were observed in the visuospatial working memory task as well as in the updating task [*t*(66) = 0.912, *p* = 0.040, *η_p_* ^2^ = 0.062, *t*(66) = 13.161, *p* = 0.000, *η_p_* ^2^ = 0.247, respectively] with monolingual children outperforming the monoliterate bilingual ones.

**Table 2:** Participants’ performance on all tasks.

	Language group		Differences		
	Bilinguals	Monolinguals			
	Mean (*S.D*.)	Mean (*S.D*.)			
Measure	*N* = 35	*N* = 35	*t*-test	*p* value	*Bayes factor*
Greek vocabulary	36.25 (7.58)	38.93 (5.58)	3.033 *ns*	0.101	5.5
Nonverbal intelligence	25.05 (0.62)	26.69 (0.68)	0.132 *ns*	0.81	5.7
Albanian vocabulary	47.00 (7.76)	-	-	-	4.8
Verbal working memory	11.91 (0.64)	11.78 (0.14)	25.000 *ns*	0.184	5.2
Visuospatial working memory	11.82 (0.75)	13.78 (0.53)	0.912*	**0.040**	5.8
Updating	35.4 (3,53)	53.27 (1.78)	13.161***	**0.000**	5.3
SR					
Grammaticality	31.22 (0.12)	31.55 (0.13)	0.085 *ns*	0.126	4.9
Accuracy	47.82 (3.63)	80.66 (1.82)	15.009***	**0.048**	5.1

S.D., standard deviation. ^*^p < 0.05; ^**^p < 0.01; ^***^p < 0.001.

Regarding the grammaticality scores in SR, no differences were detected between the two groups [*t*(66) =0.085, *p* = 0.126, *η_p_* ^2^ = 0.035]. On the other hand, the two groups differed in accuracy scores, with monolingual children performing better than their monoliterate bilingual peers [*t*(66) = 15.009, *p* = 0.048, *η_p_* ^2^ = 0.076].

In order to explore the relationship among all tasks a series of Pearson moment correlations were run. The analyses of monolingual children revealed that accuracy in the sentence repetition task correlated significantly with the visuospatial working memory (*r*(34) = 0.285, *p* = 0.032), updating task (*r*(34) = 0.528, *p* = 0.001), Greek vocabulary (*r*(34) = 0.234, *p* = 0.037), and age (*r*(34) = 0.219, *p* = 0.040; see Table 3).

**Table 3:** Pearson’s moment correlations among all variables in monolingual children.

Measure	1	2	3	4	5	6	7	8
1. Verbal working memory	-							
2. Visuospatial working memory	0.246*	-						
3. Updating	−0.160	0.102	-					
4. SR Grammaticality	−0.203	0.047	0.049	-				
5. SR Accuracy	0.061	0.285*	0.528**	0.177	-			
6. Greek vocabulary	0.235	0.065	0.088	0.083	0.234*	-		
7. Nonverbal intelligence	−0.047	−0.026	0.031	0.114	0.152	0.182	-	
8. Age	0.112	0.235*	0.283*	0.183	0.219*	0.235*	244*	-
9. SES	0.072	0.069	0.083	0.088	0.179	0.065	0.182	0.095

^*^p < 0.05; ^**^p < 0.01; ^***^p < 0.001.

The analyses of monoliterate bilingual children revealed that accuracy in the sentence repetition task correlated significantly with Greek vocabulary (*r*(34) = 0.285, *p* = 0.029), and age (*r*(34) = 0.203, *p* = 0.029; see Table 4).

**Table 4:** Pearson’s moment correlations among all variables in monoliterate bilingual children.

Measure	1	2	3	4	5	6	7	8	9	10	11	12
1. Verbal working memory	-											
2. Visuospatial working memory	0.232*	-										
3. Updating	−0.149	0.121	-									
4. SR grammaticality	−0.197	0.042	0.051	-								
5. SR Accuracy	0.049	0.053	0.122	0.131	-							
6. Greek vocabulary	0.221	0.062	0.078	0.079	0.285*	-						
7. Nonverbal intelligence	−0.044	−0.033	0.029	0.104	0.133	0.129	-					
8. Age	0.118	0.247*	0.294*	0.137	0.203*	0.275*	293*	-				
9. SES	0.058	0.063	0.077	0.084	0.122	0.134	0.088	0.075	-			
10. Home language history (Greek)	0.073	0.081	0.063	0.284*	0.105	0.113	0.142	0.093	−0.153	-		
11. Current language use (Greek)	−0.123	0.122	0.078	0.125	0.088	0.072	0.113	0.098	0.113	0.210*	-	
12. Early literacy practices (Greek)	0.068	0.085	0.074	0.293*	0.112	0.087	0.154	0.088	0.075	0.209*	0.293*	-
13. Albanian vocabulary	0.118	0.069	0.071	−0.083	−0.097	−0.064	0.101	−0.087	−0.085	−0.076	−0.073	0.082

^*^p < 0.05; ^**^p < 0.01; ^***^p < 0.001.

To explore which factors explain the participants’ performance on accuracy scores, we ran regression models with stepwise backward elimination, one for the monolingual children and one for the monoliterate bilingual children. Predictors for the dependent measure of accuracy were entered according to the significance of the correlational analyses (see Tables 5 and 6). The results of monolingual children show that visuospatial working memory and updating were the only significant predictor variables for accuracy *R* ^2^ = 0.311, *F*(1,35) = 6.582, *p* = 0.007; *β*_1_ = 0.526, *p*_1_ < 0.001 and *β*_2_ = 0.343, *p*_2_ = 0.011, respectively (see Table 5). Greek vocabulary is also shown to be a marginally significant predictor of the monolingual children’s performance in SR accuracy.

**Table 5:** Output of regression models (beta coefficients) in accuracy (SR) of monolingual children.

Independent predictors	SR accuracy
Verbal working memory	*β* = 0.014, *p* = 0.896
Visuospatial working memory	*β* = 0.526, *p* < 0.001
Updating	*β* = 0.343, *p* = 0.011
SR grammaticality	*β* = −0.028, *p* = 0.803
Greek vocabulary	*β* = 0.215, *p* = 0.054
Nonverbal intelligence	*β* = 0.120, *p* = 0.246
Age	*β* = 0.227, *p* = 0.058
SES	*β* = 0.109, *p* = 0.203

**Table 6:** Output of regression models (beta coefficients) in accuracy (SR) of monoliterate bilingual children.

Independent predictors	SR accuracy
Verbal working memory	*β* = 0.011, *p* = 0.965
Visuospatial working memory	*β* = 0.097, *p* = 0.674
Updating	*β* = 0.106, *p* = 0.611
SRT grammaticality	*β* = 0.010, *p* = 0.968
Greek vocabulary	*β* = 0.432, *p* = 0.011
Nonverbal intelligence	*β* = 0.124, *p* = 0.587
Age	*β* = 0.048, *p* = 0.844
SES	*β* = 0.153, *p* = 0.421
Home language history (Greek)	*β* = −0.436, *p* = 0.368
Current language use (Greek)	*β* = 0.003, *p* = 0.992
Early literacy practices (Greek)	*β* = 0.418, *p* = 0.354
Albanian vocabulary	*β* = 0.402, *p* = 0.311

We followed the same procedure for the monoliterate bilingual children. The results demonstrate that only Greek vocabulary predicts accuracy scores in the Greek SR [*R* ^2^ = 0.425, *F*(1,35) = 7.231, *β* = 0.432, *p* = 0.011; see Table 6].

## Discussion

The present study compares the performance of Albanian-Greek bilingual children, monoliterate in Greek, with monolingual Greek children in a Greek SR task. Bilingual participants were profiled in terms of their age, SES, language history and language practices in both languages, and their vocabulary skills in L1 and L2. All participants were assessed on cognitive tasks in order to investigate the contribution of vocabulary, cognitive abilities and language experience in the case of the bilingual children, on SR performance. The two groups were matched in terms of Greek vocabulary and the monoliterate bilingual children were balanced in terms of their proficiency skills across the two languages as shown by their vocabulary scores in each language. Based on our previous studies (Andreou, 2015; Andreou et al., 2020b), we expected that monoliterate bilingual children will perform similarly to their monolingual peers in cognitive and SR measures. Results show that the two groups had similar performance in the grammaticality scores of the SR task. This is not surprising, since the bilingual group was dominant in Greek based on their language experience measures as well as the fact that they were immersed in Greek throughout their schooling. It was also observed that monolinguals outperformed the monoliterate bilinguals in terms of SR accuracy, visuospatial working memory, and updating.

Starting with the outcomes of the cognitive tasks, recall that our hypothesis was that no bilingual advantage would be found. Specifically, our earlier studies (Andreou et al., 2020b; Andreou et al., submitted; Dosi et al., 2016) showed that the bilingual advantage was associated with biliterate bilinguals with a certain balance in L1 and L2 literacy exposure. Although we did not expect an advantage in our bilingual group, the findings still do not support our hypothesis of no difference from monolinguals; instead, an advantage is found in the opposite direction, whereby the monolingual children outperform the bilingual group in the two non-verbal memory tasks, Mr. X and 2-back. Although we do not have a direct explanation for this contrast between the similar performance of the two groups in the verbal tasks but not in the non-verbal ones, we would like to consider a couple of possibilities. First, the difference in the domain of working memory tested (verbal vs. nonverbal) may reflect a difference in the degree of familiarity that children have with the stimuli. Apart from the fact that the curriculum in Greek schools is heavily language-biased in that it relies heavily on textbooks and rote-learning (Flory and Perkins, 1984), children’s familiarity with digits compared to visuospatial tasks may also lead to a flattening of possible working memory differences in these, less familiar, domains. This possibility is further supported by the fact that the two groups did not differ in Greek vocabulary skills, despite the monolingual-bilingual distinction. We should mention here that the task employed to tap participants’ visuospatial working memory is specifically sensitive to WM’s capacity, as it demands from children to hold in mind positions in space for a short period of time and simultaneously to process this information. It was particularly selected because it is free from verbal and cultural characteristics and constitutes a good and widely used task to estimate visuospatial working memory. We should keep in mind that the children of our study are bilinguals and that any verbal task is biased toward one of their languages (dominant language), which actually shows the close link between SR and the visuo-spatial working memory task we administered here. Additionally, for assessing the children’s verbal working memory, we employed the digit backwards task, a test that requires children to hold digits in mind for a short period of time and simultaneously process this information. We took into account that the digits are verbal information and carefully chosen to minimize support from long-term knowledge and merely tap working memory. Nevertheless, the information is verbal and is less sensitive than any visual information to tap the children’s ability to process information in WM. In terms of the group difference in the updating task, we propose that the higher complexity of the 2-back task compared to Mr. X is responsible for the effects found. The updating task requires constant activation of monitoring and updating skills, which makes this task the most demanding of the three, according to studies that have found a bilingual advantage in complex, higher order tasks (Costa et al., 2008, 2009; Martin-Rhee and Bialystok, 2008). In this respect, the results are consistent with those found in Andreou et al. (submitted). In this study, 65 primary school-age Greek–German bilingual children (10–12 years old) were tested on three executive function tasks, differing in complexity. It was only updating, which was the most challenging of the cognitive tasks administered, that revealed differences among bilingual children who otherwise varied in terms of balanced exposure to bilingual education and biliteracy.

Turning to the differences found in SR accuracy scores, whereby monolinguals outperform the monoliterate bilingual group, our findings appear problematic especially in view of the groups’ similar scores in Greek vocabulary and grammaticality in SR. Two reasons may be responsible for this difference in SR accuracy. The first has to do with SR tasks drawing on cognitive resources, specifically memory, and the second with the bilingual children’s exposure to Greek.

As highlighted in the Introduction, the measure of accuracy in SR taps on both language and cognitive resources. In other words, SR is not only a benchmark of language but also of verbal working memory capacity. As shown independently of SR tasks, verbal working memory is linked to and further enhances visuospatial and updating skills (Yue et al., 2008; McVeigh et al., 2019), in that these skills draw on cognitive mechanisms that are associated with or demand working memory capacity for storing visual and verbal data. Thus, boosting one end of the working memory system may enhance the other. If our rationale for the lack of a difference in verbal working memory scores between monolingual and bilingual children presented above is correct, and the interdependence among memory tasks is also valid, we suggest that non-verbal cognitive skills, which are challenged in this group of bilingual children when compared to monolinguals, affect SR accuracy. An additional explanation may have to do with the Greek language input the bilingual children receive at home. Recall that the group of monoliterate bilingual children in this study consists of children whose parents are Albanian and who, regardless of their heritage language, decided to use only Greek as the language of communication with their children. This decision is quite common among many immigrant families (Maligkoudi, 2010) and is particularly common when the heritage language is of low prestige among the community, as is the case with Albanian in Greece. It is therefore likely that the linguistic input those children received in Greek may have not been native-like in terms of syntactic complexity and diversity. As noted in several studies, variation in the input quality and quantity that heritage speakers receive, in combination with the impact of the lack of education in the heritage language can have an effect on the resulting grammar (Rothman, 2009).

Turning to further factors, we note that positive correlation between children’s age and cognitive skills was found. As indicated in several studies, age has an impact on cognitive abilities (Harris and Deary, 2011; Xu et al., 2013; Tucker-Drob et al., 2014). Specifically, structural cognitive patterns undergo significant modulation with age progression, which is evident in the performance of children and adolescents in cognitive tasks (Mungas et al., 2013; Brydges et al., 2014).

Our second research question had to do with identifying the variables that best predict performance on SR accuracy for monolingual and monoliterate bilingual children. We hypothesized that monoliterate bilingual and monolingual children will rely on the same cognitive and linguistic mechanisms, for the SR task. Our results do not confirm our hypothesis. Specifically, our findings show that updating, visuospatial working memory and vocabulary were variables that predicted the monolingual children’s accuracy scores, with the cognitive tasks being the strongest predictors accounting for 31.1 and 34.3, respectively, of the % of variance in our data. In contrast, Greek vocabulary was the only significant predictor for the performance of the bilingual children in SR accuracy. In order to address this discrepancy in the predictors of SR accuracy in the two groups, we consider the differences attested between monolinguals and bilinguals in the cognitive tasks. Recall that the bilinguals’ strength was found in Greek vocabulary, while non-verbal working memory tasks were more challenging for them than for the monolingual children. In previous studies from the same large project cohort of bilingual children, SR accuracy scores were shown to be predicted mainly by updating and Greek vocabulary (see Andreou, 2015; Andreou et al., 2020b). Crucially, in these studies, higher scores in updating in particular were associated with a good balance in literacy exposure for both languages of the child. It is therefore possible, albeit speculative, that in the current study the lack of literacy in the bilingual children’s heritage language may be related to the attested underperformance on nonverbal cognitive tasks and on SR accuracy. The fact however that it was Greek vocabulary, rather than cognitive tasks, that was the only significant predictor of bilingual children’s performance in SR accuracy, indicates that working memory resources, which can and are drawn upon in SR tasks, are not employed by this group of bilinguals. Thus, in the face of SR demands, children rely on their language proficiency instead of their lower memory resources, as a means of performing in the SR task.

Current results are in accordance with previous research, which suggests that it is not bilingualism per se that confers a cognitive advantage. Instead, factors such as schooling and biliteracy, input in the first and the second language, and the strength of cognitive and language skills play an important role in profiling bilingual language experience.

## Limitations of the Study

In the present study the absence of an advantage (or the presence of the disadvantage) in the case of monoliterate children might be due to many other confounding variables. Factors such as lower family income (Balladares et al., 2016), minor educational resources (Piller and Gerber, 2018; in both languages) may account for this difference. In future work we aim to expand the sample by adding a group of biliterate bilingual children. Present research so far has provided some indication that bilingualism alone is not an adequate factor to predict cognitive advantage, but it is rather literacy in both languages that enhances cognitive flexibility. It is likely that this study also points towards that direction although future research is necessary to arrive at firm conclusions.

## Data Availability Statement

The raw data supporting the conclusions of this article will be made available by the authors, without undue reservation.

## Ethics Statement

The studies involving human participants were reviewed and approved by Ministry of Education of Greece. Written informed consent to participate in this study was provided by the participants’ legal guardian/next of kin.

## Author Contributions

MA, IT, EM, and EA: conceptualization, writing—review and editing. MA: formal analysis, data curation, and writing—original draft preparation. All authors contributed to the article and approved the submitted version.

### Conflict of Interest

The authors declare that the research was conducted in the absence of any commercial or financial relationships that could be construed as a potential conflict of interest.

## References

[ref2] Alloway T. P. (2007). Working memory, reading, and mathematical skills in children with developmental coordination disorder. J. Exp. Child Psychol. 96, 20–36. 10.1016/j.jecp.2006.07.002, PMID: 17010988

[ref3] Alloway T. P. Gathercole S. E. (2005). The role of sentence recall in reading and language skills of children with learning difficulties. Learn. Individ. Differ. 15, 271–282. 10.1016/j.lindif.2005.05.001

[ref4] Alloway T. P. Gathercole S. E. Kirkwood H. Elliott J. (2009). The working memory rating scale: a classroom-based behavioral assessment of working memory. Learn. Individ. Differ. 19, 242–245. 10.1016/j.lindif.2008.10.003

[ref5] Alloway T. P. Gathercole S. E. Willis C. Adams A. -M. (2004). A structural analysis of working memory and related cognitive skills in young children. J. Exp. Child Psychol. 87, 85–106. 10.1016/j.jecp.2003.10.002 14757066

[ref158] Andreou M. (2015). The effects of bilingualism on verbal and non verbal cognition: the micro- and macro-structure of narratives in the weak and the dominant language of the bilingual child. dissertation thesis. Aristotle University of Thessaloniki.

[ref6] Andreou M. Dosi I. Papadopoulou D. Tsimpli I. M. (2020b). “Heritage and non-heritage bilinguals: the role of biliteracy and bilingual education” in Lost in transmission: The role of attrition in heritage language development. eds. Brehmer B. Treffers-Daller J. (Amsterdam: John Benjamins), 172–196.

[ref7] Andreou M. Tsimpli I. M. (2020). Bilingualism, biliteracy and syntactic complexity: The role of crosslinguistic influence and cognitive skills. Language acquisition, processing and bilingualism: Selected Papers from the Romance Turn VII, 132.

[ref8] Andreou M. Tsimpli I. M. Durrleman S and, Peristeri E. (2020a). Theory of mind, executive functions and syntax in bilingual children with autism spectrum disorder. *Languages* (Special Issue on ‘Atypical speech, language and communication development’).

[ref153] Armon-Lotem S. de Jong J. Meir N. (eds.) (2015). Methods for assessing multilingual children: Disentangling bilingualism from language impairment (Bristol, UK: Multilingual Matters).

[ref9] Baddeley A. (2012). Working memory: theories, models, and controversies. Annu. Rev. Psychol. 63, 1–29. 10.1146/annurev-psych-120710-100422, PMID: 21961947

[ref10] Baddeley A. D. Hitch G. J. Allen R. J. (2009). Working memory and binding in sentence recall. J. Mem. Lang. 61, 438–456. 10.1016/j.jml.2009.05.004

[ref11] Balladares J. Marshall C. Griffiths Y. (2016). Socio-economic status affects sentence repetition, but not non-word repetition, in Chilean preschoolers. First Lang. 36, 338–351. 10.1177/0142723715626067

[ref12] Bayliss D. M. Jarrold C. Gunn D. M. Baddeley A. D. (2003). The complexities of complex span: explaining individual differences in working memory in children and adults. J. Exp. Psychol. Gen. 132, 71–92. 10.1037/0096-3445.132.1.71, PMID: 12656298

[ref13] Bialystok E. (1986). Factors in the growth of linguistic awareness. Child Dev. 57, 498–510. 10.2307/1130604

[ref14] Bialystok E. (1999). Cognitive complexity and attentional control in the bilingual mind. Child Dev. 70, 636–644. 10.1111/1467-8624.00046

[ref15] Bialystok E. Craik F. I. (2010). Cognitive and linguistic processing in the bilingual mind. Curr. Dir. Psychol. Sci. 19, 19–23. 10.1177/0963721409358571

[ref16] Bialystok E. Craik F. I. Green D. W. Gollan T. H. (2009). Bilingual minds. Psychol. Sci. Public Interest 10, 89–129. 10.1177/1529100610387084 26168404

[ref17] Bialystok E. Martin M. M. (2004). Attention and inhibition in bilingual children: evidence from the dimensional change card sort task. Dev. Sci. 7, 325–339. 10.1111/j.1467-7687.2004.00351.x, PMID: 15595373

[ref18] Brydges C. R. Fox A. M. Reid C. L. Anderson M. (2014). The differentiation of executive functions in middle and late childhood: a longitudinal latent-variable analysis. Intelligence 47, 34–43. 10.1016/j.intell.2014.08.010

[ref19] Carroll J. B. (1994). An alternative, thurstonian view of intelligence. Psychol. Inq. 5, 195–197.

[ref20] Chatzidaki A. Maligkoudi C. (2013). Family language policies among Albanian immigrants in Greece. Int. J. Biling. Educ. Biling. 16, 675–689. 10.1080/13670050.2012.709817

[ref21] Chiat S. Armon-Lotem S. Marinis T. Polisenska K. Roy P. Seeff-Gabriel B. (2013). “The potential of sentence imitation tasks for assessment of language abilities in sequential bilingual children” in Issues in the assessment of bilinguals. ed. Mueller-Gathercole V. (UK: Mulitlingual Matters), 56–89.

[ref22] Chondrogianni V. Andreou M. Nerantzini M. Varlokosta S. Tsimpli I. M. (2013). The Greek Sentence Repetition Task. COST Action IS0804.

[ref24] Costa A. Hernández M. Costa-Faidella J. Sebastián-Gallés N. (2009). On the bilingual advantage in conflict processing: now you see it, now you don’t. Cognition 113, 135–149. 10.1016/j.cognition.2009.08.001, PMID: 19729156

[ref156] Costa A. Hernández M. Sebastián-Gallés N. (2008). Bilingualism aids conflict resolution: evidence from the ANT task. Cognition 106, 59–86. 10.1016/j.cognition.2006.12.013, PMID: 17275801

[ref25] Dosi I. Papadopoulou D. Tsimpli I. M. (2016). “Linguistic and cognitive factors in Elicited Imitation Tasks: a study with mono-and biliterate Greek- Albanian bilingual children” in *Proceedings of the 40th annual Boston University Conference on Language Development*; November 13–15, 2015, 101–115.

[ref26] Ebert K. D. (2014). Role of auditory non-verbal working memory in sentence repetition for bilingual children with primary language impairment. Int. J. Lang. Commun. Disord. 49, 631–636. 10.1111/1460-6984.12090 24894308 PMC4458063

[ref154] Engel de Abreu P. (2011). Working memory in multilingual children: is there a bilingual effect? Memory 19, 529–537. 10.1080/09658211.2011.590504, PMID: 21864216

[ref27] Ensminger M. E. Fothergill K. (2003). “A decade of measuring SES: what it tells us and where to go from here” in Socioeconomic status, parenting and child development. eds. Bornstein M. H. Bradley R. H. (Mahwah, NJ: Erlbaum), 13–28.

[ref28] Flory M. B. Perkins J. B. (1984). Rote learning or real learning? Teaching a course in etymology. Class. J. 79, 340–346.

[ref29] Harris S. E. Deary I. J. (2011). The genetics of cognitive ability and cognitive ageing in healthy older people. Trends Cogn. Sci. 15, 388–394. 10.1016/j.tics.2011.07.004, PMID: 21840749

[ref30] Hernández M. Martin C. D. Barceló F. Costa A. (2013). Where is the bilingual advantage in task-switching? J. Mem. Lang. 69, 257–276. 10.1016/j.jml.2013.06.004

[ref31] Hoff E. Laursen B. Tardif T. (2002). “Socioeconomic status and parenting” in Handbook of parenting: Biology and ecology of parenting. 2nd Edn. Vol. 2 ed. Bornstein M., (Mahwah, NJ: Erlbaum), 231–252.

[ref32] Jefferies E. Ralph M. A. L. Baddeley A. D. (2004). Automatic and controlled processing in sentence recall: the role of long-term and working memory. J. Mem. Lang. 51, 623–643. 10.1016/j.jml.2004.07.005

[ref33] Kaltsa M. Prentza A. Tsimpli I. M. (2019). Input and literacy effects in simultaneous and sequential bilinguals: the performance of Albanian-Greek speaking children in sentence repetition. Int. J. Biling. 24, 159–183. 10.1177/1367006918819867

[ref34] Kapia E. Kananaj A. (2013). The Albanian vocabulary instrument. Tiranë: Center for Albanian Studies, Institute of Linguistics and Literature.

[ref35] Klem M. Melby-Lervåg M. Hagtvet B. Lyster S. A. H. Gustafsson J. E. Hulme C. (2015). Sentence repetition is a measure of children's language skills rather than working memory limitations. Dev. Sci. 18, 146–154. 10.1111/desc.12202, PMID: 24986395 PMC4309482

[ref37] Kostoulas A. Motsiou E. (2020). Family language policy in mixed-language families: an exploratory study of online parental discourses. Int. J. Biling. Educ. Biling. 1–13. 10.1080/13670050.2020.1715915

[ref38] Leikin M. Schwartz M. Share D. L. (2010). General and specific benefits of bi-literate bilingualism: a Russian–Hebrew study of beginning literacy. Read. Writ. 23, 269–292. 10.1007/s11145-009-9210-x

[ref157] Martin-Rhee M. M. Bialystok E. (2008). The development of two types of inhibitory control in monolingual and bilingual children. Biling. Lang. Cogn. 11, 81–93. 10.1017/S1366728907003227

[ref39] Mattheoudakis M. Chatzidaki A. Maligkoudi C. Agathopoulou E. (2016). Family and school language input: their role in bilingual children’s vocabulary development. J. Appl. Ling, 31, 49–69.

[ref40] McVeigh C. Wylie J. Mulhern G. (2019). Verbal and visuospatial working memory in immersion-educated bilingual children. Int. J. Biling. Educ. Biling. 22, 505–517. 10.1080/13670050.2016.1271769

[ref41] Meir N. Walters J. Armon-Lotem S. (2015). Disentangling SLI and bilingualism using sentence repetition tasks: the impact of L1 and L2 properties. Int. J. Biling. 20, 421–452. 10.1177/1367006915609240

[ref152] Moll K. Hulme C. Nag S. Snowling M. J. (2013). Sentence repetition as a marker of language skills in children with dyslexia. Appl. Psycholinguist. 36, 1–19. 10.1017/S0142716413000209

[ref44] Mungas D. Widaman K. Zelazo P. D. Tulsky D. Heaton R. K. Slotkin J.. (2013). VII. NIH toolbox cognition battery (CB): factor structure for 3 to 15 year olds. Monogr. Soc. Res. Child Dev. 78, 103–118. 10.1111/mono.12037, PMID: 23952205 PMC3950958

[ref45] Paap K. R. Johnson H. A. Sawi O. (2015). Bilingual advantages in executive functioning either do not exist or are restricted to very specific and undetermined circumstances. Cortex 69, 265–278. 10.1016/j.cortex.2015.04.014, PMID: 26048659

[ref47] Piller I. Gerber L. (2018). Family language policy between the bilingual advantage and the monolingual mindset. Int. J. Biling. Educ. Biling. 1–14. 10.1080/13670050.2018.150322729354017

[ref150] Polišenská K. Chiat S. Roy P. (2015). Sentence repetition: what does the task measure? Int. J. Lang. Commun. Disord. 50, 106–118. 10.1111/1460-6984.12126, PMID: 25208477

[ref48] Poll G. H. Miller C. A. Mainela-Arnold E. Adams K. D. Misra M. Park J. S. (2013). Effects of children’s working memory capacity and processing speed on their sentence imitation performance. Int. J. Lang. Commun. Disord. 48, 329–342. 10.1111/1460-6984.12014, PMID: 23650889

[ref49] Potter M. C. Lombardi L. (1990). Regeneration in the short-term recall of sentences. J. Mem. Lang. 29, 633–654.

[ref50] Pratt A. S. Peña E. D. Bedore L. M. (2020). Sentence repetition with bilinguals with and without DLD: differential effects of memory, vocabulary, and exposure. Biling. Lang. Cogn. 1–14. 10.1017/s1366728920000498

[ref155] Raven J. C. (1995). Coloured progressive matrices (CPM). Oxford, UK: Oxford Psychologists Press.

[ref151] Riches N. G. (2012). Sentence repetition in children with specific language impairment: an investigation of underlying mechanisms. Int. J. Lang. Commun. Disord. 47, 499–510. 10.1111/j.1460-6984.2012.00158.x, PMID: 22938061

[ref52] Rothman J. (2009). Understanding the nature and outcomes of early bilingualism: romance languages as heritage languages. Int. J. Biling. 13, 155–163. 10.1177/1367006909339814

[ref54] Simón-Cereijido G. (2017). “Sentence repetition in typical and atypical Spanish-speaking preschoolers who are English languagelearners” in Language development and disorders in Spanish-speaking children. eds. Auza A. Schwartz R. G. (Amsterdam, Netherlands: Springer), 205–216.

[ref55] Simón-Cereijido G. Méndez L. I. (2018). Using language-specific and bilingual measures to explore lexical-grammatical links in young Latino dual-language learners. Lang. Speech Hear. Serv. Sch. 49, 537–550. 10.1044/2018_LSHSS-17-0058 29625426

[ref56] Stokes S. F. Wong A. Fletcher P. Leonard L. B. (2006). Nonword repetition and sentence repetition as clinical markers of specific language impairment: the case of Cantonese. J. Speech Lang. Hear. Res. 49, 219–236. 10.1044/1092-4388(2006/019), PMID: 16671840

[ref57] Thordardottir E. Brandeker M. (2013). The effect of bilingual exposure versus language impairment on nonword repetition and sentence imitation scores. J. Commun. Disord. 46, 1–16. 10.1016/j.jcomdis.2012.08.002, PMID: 23021785

[ref58] Tucker-Drob E. M. Briley D. A. Starr J. M. Deary I. J. (2014). Structure and correlates of cognitive aging in a narrow age cohort. Psychol. Aging 29, 236–249. 10.1037/a0036187, PMID: 24955992 PMC4067230

[ref59] Vogindroukas I. Protopapas A. Sideridis G. (2009). Experiment on the expressive vocabulary (Greek version of Renfrew word finding vocabulary test). Chania, Crete: Glafki.

[ref60] von Bastian C. C. Souza A. S. Gade M. (2016). No evidence for bilingual cognitive advantages: a test of four hypotheses. J. Exp. Psychol. Gen. 145, 246–258. 10.1037/xge0000120, PMID: 26523426

[ref61] Willis C. S. Gathercole S. E. (2001). Phonological short-term memory contributions to sentence processing in young children. Memory 9, 349–363. 10.1080/09658210143000155, PMID: 11594357

[ref62] Xu F. Han Y. Sabbagh M. A. Wang T. Ren X. Li C. (2013). Developmental differences in the structure of executive function in middle childhood and adolescence. PLoS One 8:e77770. 10.1371/journal.pone.0077770, PMID: 24204957 PMC3812181

[ref63] Yue Z. Zhang M. Zhou X. (2008). Updating verbal and visuospatial working memory: are the processes parallel? Chin. Sci. Bull. 53, 2175–2185. 10.1007/s11434-008-0299-0

[ref64] Ziethe A. Eysholdt U. Doellinger M. (2013). Sentence repetition and digit span: potential markers of bilingual children with suspected SLI? Logoped. Phoniatr. Vocol. 38, 1–10. 10.3109/14015439.2012.664652, PMID: 22414332

